# The organizational challenges of municipal call centers as a health service in Norway-a multiple case study

**DOI:** 10.1186/s12913-025-12264-0

**Published:** 2025-01-20

**Authors:** Linda Grøndal-Eeles, Janne Dugstad, Hilde Eide, Etty Nilsen

**Affiliations:** Centre for Health and Technology, Faculty of Health and Social science, University of South-Eastern Norway, Drammen, Norway

**Keywords:** Health technology, Call center, Municipality, Organizing, Telecare, Telehealth

## Abstract

**Background:**

To maintain sustainability in the health care system, technology such as social alarms and sensors has been implemented in people’s homes with the goal of increasing independent living for elderly and multimorbid health care recipients. When implementing technology, someone needs to monitor and answer the alarms and calls, which is often coined ‘telecare’. Many countries have organized telecare service in call centers, which in the health care domain is a service innovation. This study aims to research how call centers in Norway were organized, what services they offered, and what challenges they faced.

**Method:**

This was an explorative study, using multiple case methodology. The study included five call centers, covering approximately 60 municipalities across Norway. 11 interviews with 15 informants, holding a variety of positions, such as managers, assistant managers, health personnel, technicians, advisors, and subject coordinators, call center observations and document studies were conducted. The data was analyzed inductively, and empirical literature as well as a framework for service innovation were used as theoretical perspectives.

**Results:**

Four types of organizational structures of call centers were identified: 1) call center combined with emergency room; 2) call center combined with other technology; 3) call center combined with ambulant team and 4) call center combined with an advisory department. One factor for innovation success has been identified as market conditions which are expected to be increasing, whilst the study identified several challenges, for example the complexity of stakeholders. Based on different stakeholder worldviews, the various methods of innovation and organization have led to a variation in services.

**Conclusions:**

Stakeholders with different worldviews, service innovation strategies and municipal autonomy have influenced how the call centers have developed in different directions. They are still in a service innovation phase, implementing new services and technology. The call centers appeared to be ‘caught between a rock and a hard place’ – situated between health and social care, but slowly moving towards acute and contingency services, that is, from telecare towards telehealth.

**Supplementary Information:**

The online version contains supplementary material available at 10.1186/s12913-025-12264-0.

## Introduction

There has been a rapid increase in the use of health technology, and recently health technology has been developed and implemented to help people live at home as long as possible, maintaining their independence and managing their own health issues [1, 2]. By 2006, about 1.5 million people were receiving telecare services in the UK [3]. However, Fisk et al. [4] indicate that there has only been a slight increase in telecare in the UK thereafter, and 2020 estimates show 1.7 million users of telecare and a reduction in services. The reduction is mostly explained by economy as it is costly to replace outdated technology. Furthermore, technology offered by the government is replaced by private technology that the public already has in their possession [4]. Another explanation is uncertainty towards how effective health technology in people’s homes is, and it has been difficult to measure economical and qualitative benefits [5, 6]. It has been difficult to measure reductions in time consumed or use of the health service after implementing health technology. A study of the British telecare service supports these findings and claims there is a shortfall between policy and evidence [5]. Further, questions are being raised regarding whether the technology is used in a way that is optimized to achieve better benefits [7, 8]. Notwithstanding, various technologies are implemented within people’s homes with the aims mentioned above [9].

Social alarms are a well-known form of technology, implemented to empower and ensure safety for, in particular, the elderly living at home [10–12]. Health technology implemented in people’s homes can submit active or passive alarms according to the needs of the users. With the advent of health technology, the need to supervise and respond to these alarms is evident. To meet this challenge, countries such as Norway, Sweden and the UK have organized call centers. A call center is a facility that is manned 24/7 with staff who respond to, assess, and document “calls” or alarms [9]. Examples of such technology can be alarms that the user physically activates when in need of help (e.g. a social alarm), or technology that automatically sends alarms (e.g. a fall sensor) [4]. Hence, call centers can be regarded as “high-tech control rooms for care” [9] and a service innovation within the health care service [13, 14].

The field of health technology and telecare is dominated by innovation and fast-moving changes. There is a lack of consensus internationally about the use of terms in the field [15, 16]. For example, the WHO [17] uses the term “telehealth” as an umbrella term and “telemedicine” as a component within telehealth. The WHO [17] defines telemedicine as: *“The delivery of health-care services where distance is a critical factor, by all health-care professionals using information and communication technologies for the exchange of valid information for diagnosis, treatment and prevention of disease and injuries all in the interests of advancing the health of individuals and their communities.”* “Telecare” is a term used by Fisk et al. [4], Hamblin et al. [18] and Procter et al. [19] when describing the service of the call center. Hamblin [20] uses the term “alarm receiving center”, whilst Procter et al. [19] use the term “call center”. In this study we use the terms “telecare” and “call center”, as these terms are well documented.

In line with the other Nordic countries (Finland, Sweden, Denmark and Iceland), Norway has a tax-financed healthcare system, where municipalities are responsible for primary care. The Norwegian Directorate of Health [21] developed recommendations for the telecare services provided by municipalities, including call center services. These recommendations were introduced simultaneously with the transition from analogue to digital social alarms and the introduction of digital platform solutions for the management of calls and alarms from a variety of health technologies installed in people’s homes [21], as well as calls from patients and users of the technological services. This is considered the starting point for the establishment of call centers in Norway as we know them today, although municipalities were working with health technology prior to this. The municipalities are not legally obligated to provide telecare services, but health technology is recommended by the authorities [21]. Currently, there is no official record of the number of call centers established, how they are organized, what services they provide, the user and patient groups concerned, or the quality of the service in Norway.

There is scant research on how call centers are organized. One exception is that of Greenhalgh et al. [22], who conclude that there is no coherent organization of call centers, and that the centers vary in organizational model and content. Despite intensive government investments in health technology, the organization of call centers is challenging. Farshchian et al. [9] describe three types of call centers. Two of the call centers can be perceived as an emergency medical dispatch and an emergency room, whilst the last type is comparable to a call center managing health technology. Both studies conclude that more research is needed in the area.

With these challenges and the lack of literature as a background, the aim of this study was to explore how call centers were organized, what services they offered and what challenges they faced.

### Health technology as service innovation – theoretical perspectives

The use of new technologies changes the way professionals work and the technology itself will play a role in the way the services are delivered [23]. An early definition of health care service innovation, organization and delivery is “a novel set of behaviors, routines, and ways of working that are directed at improving health outcomes, administrative efficiency, cost effectiveness, or users’ experience and that are implemented by planned and coordinated actions” [24]. Implementation of health technology and use of call centers is a new way of working and delivering digital health care services; it is a new practice [25], and can therefore be looked upon as service innovation.

When working with service innovation, there will be barriers and facilitators that can hinder and/or accelerate the innovation [26]. Innovation within health care encounters particular challenges, since there are no clear recipients, but rather a range of stakeholders involved [27]. To enable management of service innovation, a framework can help maintain focus through the multiple changes that an innovation may involve, especially in complex organizations such as the health care system [28–30]. De Jong et al. [13] developed a framework for service innovation by systemizing research and extant knowledge, where the core is the New Service Development process (NSD). This process can be understood as a two-step process: a search phase and an implementation phase. In the search phase, idea generation, screening, commercialization and evaluation are the main factors, whilst development, testing and launch of the service innovation are conditions in the implementation stage [13].

In addition, de Jong et al. [13] include external conditions in their framework (which are seen as unmanageable by the organization) as well as success factors (perceived to be manageable by the organization itself). External conditions are market conditions, knowledge infrastructure and government policy – either directly related to innovation success, or which may contribute to creating a supportive climate for innovation [13]. Directly related factors for success are people, structure, resources and networking, while culture and leadership, strategy, and company characteristics may contribute to create a supportive climate for innovation. Culture is at the center of an organization’s informal structure, and leaders influence culture to a high degree. De Jong et al. [13] highlight management support, open culture, internal communication and workers’ autonomy as factors for success in creating a culture that promotes innovation.

Furthermore, innovation in services comprises the following dimensions, according to de Jong et al. [13]: service concepts, client interface, delivery systems and technology, and whether the innovation is incremental or radical [13]. Incremental innovations are small changes or improvements to an existing service or product [31]. According to Miller et al. [32] “radical innovation creates dramatic change in technology, processes, products, and/or services that considerably transforms existing markets and industries, or even gives rise to new ones”. To define the actual amount of newness in an innovation can be challenging, especially service innovation, due to its intangibility [27, 33]. Most innovations in the public sector are said to be incremental [34]. However, Dugstad et al. [28] found implementation of digital monitoring technology in residential care to constitute radical innovation.

Focus on results is the final phase in the de Jong et al. [13] framework. Financial benefits, customer value and strategic success are the benefits identified in their study [13]. As De Jong et al. [13] point out, no service innovation is straight forward or linear and sometimes a trial-and-error strategy is followed, moving between different stages. Therefore, service innovation can be more difficult to manage than product innovation due to its less formal processes, which makes it more difficult to identify what stage in the process the innovators are at [35].

### Stakeholders as a means for various organizational models

Greenhalgh et al. [22] claimed that exploration of the organizational implications of increased use of telehealth and telecare lagged behind technological development. This is still true [29, 36], and the emergence of health care innovations such as the call centers under study, demonstrates the need for mapping stakeholders. Greenhalgh et al.’s [22] study is of particular interest, as it mapped four stakeholder groups with different worldviews, or contradicting ways of seeing telecare, which complicated the organizing visions for telehealth and telecare. These groups that represent different worldviews are labeled modernist, humanist, change management, and political economy groups. The modernist group was characterized as futuristic and focused on technology. The humanist group was regarded as person-centered and naturalistic. The change management group concentrated on in-house technology and supported routine. The political economy group was cautious and critical. Greenhalgh et al. [22] suggested that the stakeholder groups should establish cross-sector dialogue, and recognize and acknowledge the different worldviews, to develop a coherent vision for telehealth and telecare [22].

## Method

### Design

The study was an explorative, multiple case study [37–40]. Eisenhardt and Graebner [38] endorse multiple case studies with the argument that multiple cases generate data that is parsimonious, but also more robust. Five call centers, covering approximately 60 municipalities (of a total of 356) in Norway were explored. The data were analysed using content analysis with open coding [41].

### Data collection

The data collection was set to take place within Norwegian municipalities. In Norway, municipalities are responsible for providing primary health care services. This includes general practitioner (GP) services, emergency primary care, physiotherapy, child health clinics, school health services, nursing homes, and home care services [42]. Data collection took place in May and June 2022. Ten municipal call centers were identified in an internet search and five of these were randomly selected. The five selected call centers, located across Norway, were contacted by e-mail and agreed to participate in the study. The plan was to contact more call centers on the list if any of the invited municipal call centers declined to participate. The information on their websites describing their organization and the services provided was researched to learn how these call centers operate. Document studies and observation of operators working in the call centers were done with the aim of understanding the context and gain background information. The observations consisted of guided tours around the facilities, seeing the work area, hearing the employees describe their work and showing their workstation. Furthermore, interviews with leaders, advisors and employees were conducted as the primary data collection.

An e-mail was sent to the managers of the call centers, asking for interviews with managers, employees, advisors or others who could shed light on the topic of the organization of their call centers. The dates of the interviews were agreed with the informants, some face-to-face and some via video-link due to the pandemic.

Semi-structured interviews were conducted, with an interview guide, designed for this study, containing topics and broad, open questions under each topic (additional file 1). The idea was to let the informants speak as freely about the various topics as possible. The topics were: what is a call center?; organization; challenges and knowledge and thoughts about future call centers.

Fifteen informants with various responsibilities within the call centers, and one person who managed an IT department working closely with the call center, were interviewed. Some were interviewed individually, some in pairs or groups of three. Altogether, 11 interviews were held. The informants held a variety of positions, such as managers, assistant managers, health personnel, technicians, advisors, and subject coordinators. The majority of the 15 informants were women and three were men (see Table 1 for an overview).

**Table 1:** Overview of informants and their positions

Informant	Gender	CC	Position	Manager	Assistant Manager	Advisor	Employee	Operation technician
1	F	CC1	IT-Manager	X				
2	F	CC1	Advisor -call center			X		
3	M	CC1	Advisor -call center			X		
4	F	CC1	Call center operator/ nurse				X	
5	F	CC1	Call center operator/ nurse				X	
6	F	CC2	Call center manager	X				
7	M	CC3	IT-Manager	X				
8	F	CC3	Municipal advisor			X		
9	F	CC3	Call center operator/Nurse		X			
10	F	CC3	Assistant manager/Nurse				X	
11	F	CC4	Call center manager	X				
12	F	CC4	Advisor- call center			X		
13	F	CC5	Municipal/Manager	X				
14	M	CC5	IT/ Technician					X
15	F	CC5	Health care worker				X	
Sum				5	1	4	4	1

^*^CC Call center, *F* female, *M* male

### Analysis of the interviews

The interviews were transcribed verbatim, stored on a laptop in a classified area and analyzed in two steps using NVivo 5.0. The interviews were read and analyzed inductively, with quotes and statements being organized into codes [41]. The first author (LGE) conducted the first tentative analysis. The codes were inspired by the aim of the study and were empirically driven. Examples of codes were “Organization” and “Challenges”. As the transcriptions were read, statements that referred to the organization of call centers were placed under the “Organization” code, while statements interpreted as challenges were placed under “Challenges”. When all the interviews were coded, the team of authors held a workshop where the coded text was read and discussed, topic by topic, to confirm the results. During the workshop the author group had the opportunity to discuss each other’s views and ask critical questions. The authors have different backgrounds, and the group is multi-disciplinary. The content has therefore been investigated from different points of view, attempting to minimise the threat of personal biases.

## Results

The results are presented in two sections. Section 1 covers descriptions of the call centers included in the study, including a model and reasons for the various organizational models. In Sect. 2, results related to challenges and possibilities for current and future services are reported.

### Organization of the call centers

In line with the majority of municipal call centers, the call centers included in the study were organized as partnerships between several municipalities – legally defined as an administrative host municipality cooperation [43]. The host municipality provided the call center services on behalf of all the municipalities in each cooperation. The cooperations typically consisted of neighboring municipalities within the same county, but also provided services to municipalities in other parts of the country.

Their primary task was to answer, evaluate, and document calls and alarms from health technology instalments in service recipients’ homes, and take appropriate measures if they were unable to clarify the situation over the phone. The measures included referral of the call to the home-care service in the municipality responsible, or the emergency services when appropriate. Hence, the call centers interacted directly with the service recipients, the technologies, and the health care workers in all the municipalities on a daily basis.

The technology services varied between the five call centers and between the municipalities cooperating in each of the centers. The health technologies included indoor and outdoor social alarms; the latter combined with localization technology such as a global positioning system (GPS). Furthermore, all the centers monitored automated medicine dispensing services (robots). Some included digital monitoring services, both camera-based and sensor-based, and smart electronic door locks.

#### Description of the different call centers and organization models

Below is a description of the five call centers in terms of how many participating municipalities they cover, their organizational location (within the municipality), and the professional background of the employees.

##### Call center one

**Fig. 1:**
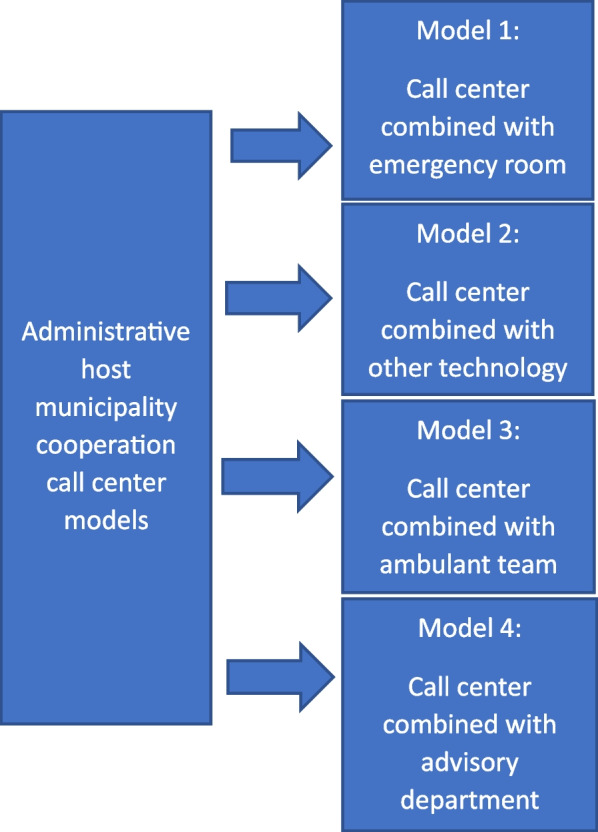
Model of administrative host municipality cooperation call center organization in Norwegian municipalities

Call center one (CC1) was organized between 14 municipalities with one municipality serving as host. CC1 was organized in the IT department within the municipal structure. It was co-located with the emergency room (model 1 in Fig. 1). Only nurses were employed. The nurses worked shifts between the two establishments. Therefore, the nurses did regular emergency room tasks when working shifts there. CC1 and the emergency room were separated by a glass wall with an open door, so the nurses at work could communicate and consult with each other.

##### Call center two

Call center two (CC2) was a collaboration between 16 municipalities. CC2 had developed from a former fire emergency dispatch and was currently organized under the health department within the municipal structure. The fire emergencies were centralized to a regional fire emergency dispatch, but the call center still monitored technology outside the health domain; i.e., safety alarms, building alarms, lift alarms and weather forecasts (model 2 in Fig. 1). CC2 was organized as a separate unit and employed nurses in addition to technicians. One nurse and one technician were always at work at the same time, and they both responded to all kinds of alarms.

##### Call center three

Call center three (CC3) was a collaboration between 16 municipalities, organized under the health department in the host municipality. CC3 was divided into two departments, where department one handled alarms from health technology as their primary task. Department two consisted of an ambulatory team that responded to service recipients in need of assistance outside planned home care service (model 3 in Fig. 1). Department two provided services in the host municipality, but not in the other municipalities in the collaboration. Only nurses were employed in CC3, and they worked shifts in both the call center and the ambulatory team.

##### Call center four

Call center four (CC4) was a collaboration between six municipalities, organized under the health department in the municipal structure. Like CC3, this call center had two departments. The first was a traditional call center service. The second was an advisory department, aiding municipalities in the collaboration regarding implementation strategies, service design, procurement, training, and technological support (model 4 in Fig. 1). In CC4 all the operators responding to calls were healthcare workers. The advisory department consisted of nurses, a teacher, an occupational therapist and healthcare workers.

##### Call center five

Call center five (CC5) was a collaboration between 12 municipalities organized under the health department in the host municipality. Like CC2, this call center developed from a former fire emergency dispatch. Nurses and health care workers were employed, and they had made demands for more nursing positions in the call center. They answered, evaluated, and documented calls related to health technologies, and took measures if they were unable to clarify the situation on the phone. In addition, CC5 handled technical alarms (in line with CC2) from several municipalities, as well as private companies. Hence, they were a model 2 call center (Fig. 1). Interestingly, they were in the process of being co-located with the emergency room, thereby transitioning to become a combination between model 2 and model 4 (Fig. 1). Furthermore, this call center had established a network for technicians in the municipalities within the collaboration. This network typically consisted of wardens in the municipalities, who also operated as technicians for the health technology.

#### Different organization models – background

Whereas four of the call centers were organized within the health department of their host municipality, one center (CC1) was under the IT department. In all the call centers included in the study, technological development was considered an important task, in addition to receiving calls and alarms. However, the call center organized within the IT department viewed its localization therein as appropriate due to the demand for innovation, whereas the health department was traditionally more focused on operation. As informant P explained:

*“Organizationally, the call center is under the IT department. We`ve fought a lot for that … because it`s a lot about technical development. And it's about safety. If it had been put under health, we were afraid that when the austerity knife begins cutbacks and savings, then innovation and development will go first, or will be some of the first to go. (…) It is under IT that development takes place, in many of the areas”.*

The leader of call center 2 (CC2) described the transition from an independent unit to being incorporated into the health department. This call center went through a transformation from being a former fire emergency dispatch to a call center in the healthcare service. Informant F gave the following details:

*“The difference is, when we moved to health, then the focus is on health. This focus is challenging when we run interdisciplinary services”.*

The informants regarded the national recommendations as lacking. This made it challenging for the call centers to define themselves and inform the public about what services they offered. They even felt they were operating a “hidden service”, as commented by informant F:

*“I think it depends on the understanding of a call center, that they do more than just safety technology, or social alarms. And it is based on the fact there are no national reports, experiences or research results that point to this, where you can read and understand what we can do. So therefore, these are “hidden services” with questions such as, “what are they doing at the call center?”*

The call centers themselves expressed a need for national standards. They were taking these initiatives to the government. Informant K explained:

*“To my knowledge, we are missing some national standards and experience on that type of call center. So, I think we lack something superior … Not just recommendations, but experience, experience-based learning, in relation to call centers under municipal auspices.”*

### Functional challenges and possibilities for current and future services

The informants felt that it would be too demanding for one municipality to find the necessary competence and funding for the operation of a call center 24/7. Therefore, organization as an administrative host municipality cooperation offered some benefits. The service needed a high number of patients and technologies to be sustainable. Informant A said:

*“The national guidelines, the one on call centers, point out that it pays to establish them, but we cannot have so many in Norway, because it is not sustainable”.*

However, only a few years ago, no one had yet developed a municipal call center. This presented challenges with the process, as Informant G explained:

*“We didn't really understand what processes we were doing because it was so new to us”.*

Another component was the lack of competence regarding technology-based services. Sharing both the workload and competence between municipalities seemed to be a more affordable solution. However, the service was relying on only a few people, and if anything happened to them, operations could potentially go wrong. Informant B described the situation:

*“What if something happens to me? A lot will then fall apart, because at that time I was the one who had much of the knowledge”.*

The main tasks of all the call centers are to receive, assess, document, and follow up alarms from health technology. Furthermore, the call centers have taken responsibility for testing technology and have built up experience and knowledge about the technology they offer to service recipients. All five call centers report managing high numbers of alarms daily. As Informant K explained:

*“We do see that we get an enormous number of alarms that go directly to our operators, but also alarms which are clarified via our automatic systems. In 2021 we received 300 000 alerts, which means that together with our automatic systems, our health operators have clarified 861 events in 24 hours, which again means 36 events per hour. Imagine if all those alarms were to be handled by the home nursing team”.*

With such numbers – and this is just one example of many – it seemed justified to have a call center. Yet some call centers explained in their interview how they were constantly defending their existence because it was not a service required by law.

#### Content of the call center service

One thing the informants agreed on was that a call center responds, documents, resolves the situation, or delegates the task to the executing services. Informant K reflected:

*“It is not self-explanatory to call it a call center, and it is in any case not self-explanatory for the general population. But for me it's about us responding to technology installed in the service recipients’ homes via our call center. As an extension, that response is up for assessment by our healthcare operators to either be clarified with us, or forwarded to emergency services, or the home service.*”

The call centers were constantly looking for new services to adopt, or ways to develop their services further. Several of the call centers explained this phenomenon with the uncertainty of whether their service “was enough”, arguing that the more services they could implement, the “safer” their existence would be. As informant F explained:

*“I think we have only just started. If we think that we are on the first step now, then we should look at digital home monitoring, which can also have many facets. But I think tasks will transfer from the general practitioners. Can the call center be a gateway into the healthcare system, and then assessments are made here, by a team? We could be the entrance for residents and next of kin. We could guide them”.*

The general expectation from the call centers was that they could all do much more than answering health technology alarms. However, one call center was content to make sure they provided high quality health services, although they did not exclude the idea of incorporating other services in the future. Some call centers were already overseeing technical alarms outside of the health care services. Examples of such alarms could be lift and theft alarms. According to informant F:

*“We are an old emergency fire dispatch. We have a portfolio of 14 services and those tasks must be solved. We still have fire, elevators and burglary alarms. We have partial responsibility for contingency and send out population alerts.”*

One difference worth noting is the choice of unique names for the call centers. Whereas some were called call centers (“response centers” in Norwegian), others had taken names decided locally. It seemed to cause confusion among the employees working with or in the call centers, as they had difficulties defining themselves. Informant F stated:

*“One thing is, what should the naming of call centers be? It can cause uncertainty externally. But what shall it be? That is probably why the municipalities have gone in different directions, as well.”*

#### Characteristics of alarms

To get an overview of the workload related to the alarms received we asked about what kinds of alarms the call centers managed: whether they received any acute alerts or alarms and, if so, how many; or whether they received mostly non-urgent questions. Informant E elaborated:

*“There are a lot of ordinary alarms. Much of the call center work doesn't require a nurse. Such as; I need help to go to the bathroom; Can someone come and close the window. It is quite a lot of these alarms.”*

Some said these non-urgent alarms amount to approximately 90% of the alarms, and possibly even higher. According to our informants there were no exact numbers. All the call centers reported various numbers of acute events, sometimes several a day. However, their occurrence was unpredictable amongst the more ordinary non-urgent alarms. Informant J revealed:

*“113 alarms? (acute alarm/call) It can be three in a day. Then a couple of days can pass before the next one.”*

Alarms requiring an urgent response seemed to be increasing. The call centers reported that they handled more at-risk patients, and that service recipients in general were more complex, having several illnesses. Apparently, the most ill service recipients were those who got health technology. The informants hoped that technology would be allocated to service recipients in the earlier stages of their illness. Informant M disclosed:

*“In our experience, we manage symptoms that are so severe that we must contact and notify the ambulance increasingly often. This is what makes us think about co-location with the emergency room. Not fully integrated, but under the same umbrella, so we are sure that we have both nurses and doctors available in complicated circumstances and to discuss cases.”*

Call centers clearly handled both acute and non-urgent cases. The alarms included everything “from A to Z”, and there was no definition as to what kind of calls the employees could expect. Therefore, they needed to be prepared for everything every time an alarm was received. They had to assess what was needed for each alarm and single out the one patient out of the hundreds that was seriously ill or in need of immediate response. Informant D expressed:

*“To clarify, what is needed (…). Many say they haven’t pushed any buttons or things like that, to the ones who sit there having a heart attack. So, there are quite a few things we are supposed to react to and treat it properly.”*

It was demanding and challenging to work at a call center, as the operators needed to be alert with each alarm they received. Only one of the call centers used a decision support tool in the form of a questionnaire to help guide the conversation. They had developed it with inspiration from another municipal call center. However, the tool said nothing about further action if the problem could not be handled over the phone. As there were no national guidelines for decision making in call centers, alarm management depended on who was at work and their experience. Informant D was an experienced nurse who had worked many years in the emergency room. She explained:

*“Take for example a heart attack. They will say they have pressure and pain in their chest and the pain will shoot up into their left shoulder and arm, they will be sweaty in their forehead and feeling sick. All of these are classic symptoms for a heart attack. I don't need any index for that. Because I know I need to send an ambulance. Then I tell them to sit down, and just be still. This is like a reflex to us experienced [staff] who have worked at the emergency room for a while. It`s all about experience.”*

As the results show, call centers handle a variety of alarms, mostly non-urgent, but with an increase in urgent alarms. Furthermore, the results reveal a high number of alarms per day in the call centers.

#### Staff

It was acknowledged by everyone that the operators in a call center must have a health care background. Some call centers only employed nurses. Others employed health care workers and other nurses and technicians. There were different opinions as to whether it needed to be nurses only, or whether these could be partly replaced by other health care workers. Informant P said:

*“The emergency room has one nurse, and we have one nurse. They are cooperating, and I think it is a good thing. So, I don't need any more nurses. I will recruit a health care worker next time. Then they have a nurse to ask it they are concerned if it is a stroke or heart attack, or whatever has happened.”*

Some call centers reported that hiring staff was a problem and keeping them another. Others reported that recruitment was not a significant challenge for them. It seemed that the call centers with combined positions struggled less with the process of hiring employees than call centers that only had positions as operators. Informant G elaborated:

*“We applied for nurses, to both the call center and the ambulant team. We wrote in the job advertisement that they would have combined positions. It was close to 100 applicants. It was many, something we were not used to”.*

## Discussion

This study has explored various organizational models within the municipalities and has identified four different ways of organizing municipal multi-host call centers. The infrastructure within the municipalities varied according to strategies, innovation characteristics, culture, and leadership models. Furthermore, these variations have led to the delivery of diverging services. The results found in this study contribute to illuminating the call centers’ progress in the new service development process and suggest why they have developed in different directions.

The results will be discussed in relation to other empirical studies (4,5,22,26,33), and supportive and challenging factors will be discussed according to de Jong´s framework of Service Innovation (13) in the following discussion.

The results shown above indicate that the municipal call centers are innovations within the municipal health care service, and that it is difficult to identify or measure the actual results mentioned in de Jong et al.’s [13] framework. These are: financial benefits, customer value and strategic success. The study has identified one success factor and several two-fold factors, as well as barriers to a call center as an innovation. The two-fold factors are both facilitators and barriers, and there appear to be more barriers than facilitators. One of the barriers is the complexity of stakeholders [22], and the discussion begins by looking at how various world views influence the way the call centers are organized and positioned within the municipalities.

### Organization of the call centers – stakeholders with contradicting worldviews

Four different models for organizing call centers were identified as well as two alternative organizational locations within the municipalities. One explanation for the variations in models is the fact that Norwegian municipalities are self-governed to a large extent. This means that the municipalities are free to organize the call centers as they choose, and to the level of their choice, provided they fulfill the main requirement [43].

Another explanation can be found in conflicting stakeholder worldviews, as laid out by Greenhalgh et al. [22]. Strong stakeholder groups with different worldviews complicate the organizing visions for telehealth and telecare in the municipalities. The dedicated people who work with and in the call centers come from different backgrounds. Some have a health service background, others a technical background or service backgrounds such as fire dispatch and IT backgrounds. These different backgrounds influence their worldviews, and these different worldviews can be and explanation for the different ways of organizing the call centers. However, this study found, the different categories identified by Greenhalgh et al. [22] were not mutually exclusive. This meant that, for instance, even if the dominant characteristic was modernist, it was possible to identify humanist features, and organizations characterized as political economist could have elements of the modernist and/or humanist.

The advisors, employees, subject coordinator, operation technicians, and managers working in the call centers included in this study, demonstrated technology optimism, exploration and futuristic conduct. They believe in the technology they handle and are interested in new technology and exploring new services to bring into the call center. They express confidence about the service they deliver. These findings are in line with the modernist worldview Greenhalgh et al. [22] identified in their study. Greenhalgh et al. [22] describe the modernist with characteristics like technology optimism, developing and implementing technological solutions and self-monitoring. The aim of the modernist is for health technology to lower costs and manage the demographic challenges – enabling people to live at home longer and new markets for health technology to boost the economy [22].

Greenhalgh et al. [22] describe world view change management as a focus on “in-house-technology”, service models, organizational routine, creating opportunities for system redesign and supporting routines [22]. This applies to the call center that developed, together with their existing supplier, a platform for their call center from the IT system that the municipality already used. This call center was also the only one to use support tools for decision making for their employees that handle the alarms. This decision support tool was self-developed, based on inspiration from another call center.

Managers of the call centers are ambitious and eager to grow and expand the call centers, including both more municipalities and services. In parallel to this, they need to cut costs, which creates conflicts, and the dominating worldview is that of political economy – particularly for the managers – with conflicts arising between interest groups [22].

The differing world views of the various stakeholders lead to a conflict of interest and may be part of the reason why the call centers turn out differently and develop in different directions.

### Call centers as innovation

Call centers represent a new way for health personnel to work and collaborate with other professionals. It is a new way of providing health services to the public and of receiving health care for service recipients. It can be defined as an innovation according to the definition of Thakur et al. [44]: “a new way of helping medical professionals work smarter, faster, better, and more cost effectively” [44]. Furthermore, as an innovation it represents a mix of the four changes identified by de Jong et al. [13]: changes in concept, client interface, delivery system, and technological options.

The call centers have features similar to the already existing 24/7 emergency services. One call center in this study is located together with the emergency room, and another soon will be. Most of the innovations in the public sector are incremental [34], and Snyder et al. [45] support Hartley and Rashman in suggesting that previous research shows most service innovations in general are incremental. However, Bolton et al. [46] indicate that even minor changes within a service concept can make massive differences. This supports labeling municipal call centers as incremental innovations, which means they are an improvement of a previous service [31].

However, to our knowledge, there was nothing like municipal call centers as we know them today in the Norwegian health care service before 2018. The call centers are a new type of health service, handling digital health technology, on a larger scale, installed in people’s homes. Furthermore, the call centers in this study distinguish themselves from emergency rooms and other emergency departments by the fact that they handle both active and passive alarms from service recipients living at home. Moreover, the emergency rooms and other emergency departments are regulated by emergency medicine regulations [47] and their service is structured by decision-making tools. This is not the case for the new call centers.

The level of newness – and newness in general, not only to a particular organization or sector – contributes to distinguish radical from incremental innovation. However, as argued by Van Poucke [33], the level of newness in an innovation can be difficult to define. Van Poucke [33] concludes that the value of an innovation is not its level of newness but rather the value that the innovation provides. In this study it is difficult to draw any conclusion with regard to the level of newness with the municipal call centers. An important finding in our study was that the lack of maturity of the call centers makes it difficult to measure results or value. The call centers themselves, however, have no doubt about the value they bring to the health care service and fear the consequences if they were to be halted. The data shows that municipalities have chosen different strategies during the innovation process. The municipalities articulated difficulties with strategic planning, since organizing call centers was new to them. Knowledge, new technology, and financing were perceived as challenges at the time.

The task of establishing call centers appears complex: what kind of people to employ – nurses, health care workers and/or technicians? / how and how many to employ? / where to place the call centers within the municipal structure. All these were strategic challenges the leaders of the call centers brought forward and led to different paths when choosing operating models, resulting, as seen, in four different organizational models. Nonetheless, the results revealed that all the call centers wanted something the other call centers had. For example, one call center, which was co-located with the emergency room, would have liked to have an ambulant team. Another call center, handling more than health alarms, would have liked to have a technical support department. This is an indication that the call centers have not yet found an optimal service concept.

### Supporting factors for service innovation in municipal call centers

De Jong et al.’s [13] framework of service innovation shows a range of success factors for service innovation in organizations: external conditions; success factors for creating a supportive climate for innovation; and success factors directly related to new service development (NSD). This success factor is market conditions, which is an external condition defined by de Jong et al. [13].

A market condition is identified as an external condition and the current situation in the health care service, where the market is expanding, has led to innovative ways to deliver health care services, which may have the potential to be sustainable for the future [1, 22]. On the other hand, as an output or result, one can ask if this is the services the public want – or if it creates customer value [13]. Although the external condition of government policy says that technology ought to be used to a higher degree, Fisk et al. [4] found only a small increase (200 000) in the period from 2006 – 2020, and they claim that it appears difficult to find good solutions to implement technology that relieve the health care system. After being in operation for five years the call centers could be expected to be focused on the results in the de Jong et al. framework [13]. Notwithstanding, it appears that the call centers have yet to reach the result phase. Nonetheless, the market conditions are there, and will increase in the future (48).

### The “two-fold” factors

Some of the factors perceived as supporting/facilitating may also be perceived as barriers, or as Thijssen et al. [26] found, overlap between the two. People form one such “two-fold” factor, identified as a success factor directly related to NSD (new service development) in de Jong et al.’s [13] framework. Dedicated people have contributed to the development, organization and implementation of the call centers, and this has been established as a success factor in other studies of implementation of innovations [28]. When the call centers were established, they played an important role for the politicians and in getting cooperation between municipalities up and running.

The results show that much responsibility for the development of call centers lies with very few people. In addition, the results indicate that there is still a lack of people. In general, this is a well-documented challenge within the health care system [48]. In other words, people are at the same time a success factor and a barrier, since there is a lack of them. This is in line with Thijssen et al. [26], who indicate human resources as a facilitator, but also point to lack of people as the most common barrier to implementation.

Another aspect to the challenge of people is the recruitment of people to work as operators, and here, the findings are contradicting. Some of the call centers have identified recruiting and maintaining their health personnel as a challenge. This is in line with the labor market situation in the health care sector in general, since there is a lack of health personnel [48]. However, some of the call centers report the opposite. That recruitment has not, to date, been a challenge. It appears that the call centers struggling to recruit and keep employees have only health personnel working in the call centers as operators. The call centers that report not having these issues have nurses working in combined positions, such as 50/50 call center and emergency room. This means that the combination of tasks and demands on proficiency and knowledge is more varied and possibly more demanding, which appears to be attractive to health care personnel. To be an attractive employer is important not only for attracting health personnel, but also for keeping them. This influences planning for knowledge and further development [49]. Recruiting and keeping health personnel is a universal problem across the health sector.

People are part of the organizational culture and are influenced by the leadership. Culture also has an impact on innovation [50], and culture and leadership are seen as success factors for a supportive climate in de Jong et al.’s [13] framework. The mere fact that the call centers were developed, organized and implemented in some municipalities, signalizes a culture and leadership that has supported service innovation. However, culture and leadership can work as a barrier in other ways. In some municipalities call centers were not established at all and in others, municipalities pulled out of ongoing call center projects. Furthermore, the municipalities with established call centers seem very much influenced by municipal politics, finance, culture and leadership within the municipalities. Culture for innovation can therefore be both a barrier and a facilitator, depending on the municipality.

Government policy is another example of a “two-fold” condition. The government issued recommendations for telecare services in 2016 [21], and these recommendations were a facilitator at the time. They are seen as the starting point for organizing municipal call centers as we know them today. On the other hand, the recommendations have not been updated since their first release in 2016. Therefore, the call centers perceive the recommendations as a barrier today because they are outdated due to new knowledge and increased experience in the area, and because technological development is advancing quickly [10].

### Challenges for service innovation in municipal call centers

To summarize, the study has identified challenges that the call centers faced, both at the time of their organization and implementation, and currently.

Various organizational models of operating have led to broad knowledge- and experience-building in the field of telecare service. Service innovations often go through a trial and error period [13], but potentially, this creates a challenge concerning equal access to healthcare– known as the equality principle in Norway [51, 52]. The various ways in which call centers are organized may result in inconsistent provision of health care services across the country and does not ensure equal access to health care. The various extra services, beyond the core service, and the variations in staff competence are examples.

“Staff” is included in the service concept [13], because staff and their knowledge will affect the service given. To provide a health service as remote health care, such as call centers, is complex and demands a certain set of skills and training [53]. In Procter et al.’s [19] study, staff explain how calls that are not 999 (emergency) calls, but still serious, are the most difficult to handle. Although the job is considered to be the same – to answer, assess, document and evaluate an alarm – the results show that call centers have lack of national standards and standardized ways to handle calls and the varying levels of staff competence and experienced, will affect the health assessments done in the call centers. Training for call center staff often not prioritized, and special skill sets are recommended [4]. The call centers researched in this study agree that it should be staff with a health care background working in call centers but consider it debatable whether it needs to be qualified nurses or personnel with shorter formal training. However, the results show that call centers that employ healthcare workers rather than nurses, would like to employ qualified nurses.

The call centers report a noticeable deterioration in people living at home, and this affects the nature of the calls, and they increasingly receive emergency calls. This result is in line with findings in the UK [4], and they see an increase of medical assessments in the call centers [19]. Institutions designed to receive emergency calls are regulated by the emergency medicine regulations in Norway [47]. Currently, call centers are not defined as emergency institutions by the government and are not regulated by the emergency medicine regulations. It appears that the call centers are falling between a health care service and an emergency service. The increase in medical assessments also requires higher competence, as discussed above.

In concluding the discussion, this study contributes to shedding light on how far the municipal call centers have reached in their development, how they have drifted in different directions, and how service innovation within the public sector – especially the health care sector, has played an important part in creating a new service. The study has also found evidence of conflicting worldviews, which is in line with the findings of Greenhalgh et al. [22] and their theory of why telecare and telehealth organization is hampered. In summary the study highlights three explanations as to why call centers have been organized and developed in different directions: self-governance of the municipalities, service innovation and worldviews.

## Limitations

The selection of municipal call centers is a limitation of the study. Based on scarce knowledge of the existing centers, the selection was random due to practical considerations. In this sampling approach there is potential for overrepresentation, where certain groups may be overrepresented (or underrepresented), and the ease of access to participants might compromise the reliability and validity of the data [54]. With regards to generalizability, the study cannot be generalized to the entire population, which is a common limitation in qualitative studies.

The study is a cross-sectional study which holds limitations to be aware of. A cross-sectional study provides a snapshot at one point in time, which may not be representative of long-term trends or behaviours.

With the use of multiple case studies, it has been possible to discover and explore the various organizational models of the call centers. This variation had not been possible to describe with a single case study [38]. However, a multiple case study is complex and can be challenging. Depth versus breadth in the data collection is, for instance one example of a trade-off that must be considered. A multiple-case design is regarded by many as more robust than the single-case design [37, 38], but the degree of data-depth is likely to suffer in a multiple case study [54], and could be seen as a weakness of the study.

## Conclusion

This study has presented results and is, to our knowledge, the first study to identify how Norwegian call centers are organized, and to map how they operate. Further research of call centers has been suggested by the literature, but it is also welcomed by the call centers. The call centers are working towards professionalizing their services and research is one step towards this goal. Indeed, using a multiple-case study has uncovered results that could not have been found researching only one call center. It has uncovered the organizational structures of four call centers, as well as different organization within the municipalities. Moreover, the study has uncovered the organizational structures of four call centers, as well as different organization within the municipalities. The municipalities` different worldviews is one explanation for this, in line with what was discovered in the UK.

That the implementation of call centers is a service innovation is supported by the literature and the findings in this study. Whether this is radical or incremental is, however, not concluded. This is because of the difficulty in measuring the results or value of the call centers due to their lack of maturity. As the development and innovation of the call centers are ongoing, the call centers have yet to reach the result phase in the innovation framework.

Market conditions – meaning a growing elderly population and medical advances – has been identified as a success factor and is one of the reasons for the implementation of call centers. Furthermore, these market conditions are expected to increase in the coming years. The largest group of conditions are the two-fold conditions that operate as both facilitators and barriers. One goal must be to eliminate the barriers and change the two-fold conditions into purely success factors. As barriers, the study has identified the lack of equality in the health care services the call centers provide, and staff competence. With the results obtained, the study identifies issues for further research and implications for practice.

### Issues for further research

This study has examined one type of call center and has only focused on the actual service given to end-users to a limited degree. This leaves at least two areas in need of further investigation. First, further research into the quality of the call center services, which could include a focus on knowledge level; second, how deeply the use of different staff affects the service given, instructions, decision-making tools and end-users.

### Implications for practice

This study offers a basis for municipalities to reconsider and evaluate how they organize their call centers. Increasingly, people continue to live at home with complex illnesses and disabilities, and it is likely that the alarms in call centers will rise in the future. eHealth is seen as a way to meet the challenges in the healthcare sector, hence organizational issues will be increasingly important.

## Supplementary Information

Supplementary Material 1.

## Data Availability

The datasets generated and analyzed during the current study are not publicly available due to privacy reasons but are available from the corresponding author on reasonable request.

## Abbreviations

CC: Call centers
EN: Etty Nilsen
HE: Hilde Eide
IT-department: Information technology department
JD: Janne Dugstad
LGE: Linda Grøndal-Eeles
SIKT: Norwegian Agency for Shared Services in Education and Research
WHO: World health organization
