# Supplementary material for: The organizational challenges of municipal call centers as a health service in Norway-a multiple case study
Source: BMC Health Serv Res. 2025 Jan 20;25:108. doi: 10.1186/s12913-025-12264-0 (PMC11749625; doi:10.1186/s12913-025-12264-0)
Supplement: Supplementary file 1 — Supplementary Material 1. [file 12913_2025_12264_MOESM1_ESM.pdf]

Interview Guide- Call Center Study

- How do you understand the word telecare in the municipality- (What is the meaning of it- in your opinion)

- How do you think the call center service is working today?

  Facilitators?

  Barriers?

- Is there anything you would change with the service? (– Especially if you think about the organising). If you have examples?

- Can the regulations/legislation hinder or facilitate the service you provide? Explain why it is a hinder- or the opposite. Examples?

- Are you delivering call center services the way you would like it to be- can you explain why- or why not?

- How do you cooperate with other health services, such as AMK (113) and emergency room?

- Have you experienced any ethical challenges, in connection to delivering telecare services? Any examples?

- Competence and knowledge- is it a challenge, or not. Examples of knowledge and competence, and challenges- if you have any.

- In what way, in your experience, is the service recipients involved in developing the service?

- How do you view the future of the call center services?

  What challenges/Facilitators will we meet?

- Do you have any thoughts to what the content of the call center services could be in the future?

- How will the future municipal call centers be organised?
